# Ameloblastic carcinoma: a clinicopathologic analysis of cases seen in a Nigerian Teaching Hospital and review of literature

**DOI:** 10.11604/pamj.2018.31.208.14660

**Published:** 2018-11-27

**Authors:** Olujide Oladele Soyele, Kehinde Emmanuel Adebiyi, Olufunlola Motunrayo Adesina, Adeola Mofolouwake Ladeji, Adetayo Aborisade, Abiodun Olatunji, Henry Ademola Adeola

**Affiliations:** 1 Department of Oral Maxillo-facial Surgery and Oral Pathology, Obafemi Awolowo University, Ile-Ife, Nigeria; 2 Department of Oral Pathology and Oral Medicine, Faculty of Dentistry, Lagos State University College of Medicine, Ikeja, Lagos, Nigeria; 3 Department of Oral and Maxillofacial Surgery and Oral Pathology, Obafemi Awolowo University Teaching Hospital Complex, Ile-Ife, Nigeria; 4 Department of Oral and Maxillofacial Pathology, Faculty of Dentistry, University of the Western Cape and Tygerberg Hospital, Cape Town, South Africa; 5 Division of Dermatology, Department of Medicine, Faculty of Health Sciences and Groote Schuur Hospital, University of Cape Town, Cape Town, South Africa

**Keywords:** Ameloblastoma, ameloblastic carcinoma, odontogenic tumors, cytological atypia, sub-saharan Africa

## Abstract

**Introduction:**

Ameloblastic carcinoma is a rare malignant odontogenic neoplasm that exhibits histological features of ameloblastoma in combination with cytological atypia. It may arise de novo or secondarily through malignant de-differentiation of pre-existing ameloblastoma or odontogenic cyst. Secondary ameloblastic carcinomas often results from repeated surgical intervention, which is a mainstay of odontogenic tumor management in resource limited settings. To date, relatively few cases of ameloblastic carcinomas have been reported and many cases have been misdiagnosed as ameloblastoma. This is due to its wide range of clinicopathological feature which range from indolent to aggressive. It may present as an aggressive ulcerated mass or as a simple cystic lesion; hence, it often challenging to delineate from its benign counterpart, ameloblastoma.

**Methods:**

this study reviewed the clinicopathological data on 157 cases of odontogenic tumors diagnosed over a 10 years period from the pathology archive of the Oral Pathology Unit of Obafemi Awolowo University Teaching Hospital Complex (OAUTHC), Ile-Ife, Nigeria.

**Results:**

of all these cases, we identified that 64.9% were Ameloblastomas, while 8.3% were ameloblastic carcinomas. Primary subtypes of ameloblastic carcinoma constituted 23.08%, while 69.23% of the cases were of the secondary subtype. We also found that the secondary subtype of ameloblastic carcinomas showed a higher mean duration value of 7.7 years. Most lesions were found in posterior mandible and presented with ulceration, perforation and ill-defined borders radiographically.

**Conclusion:**

this study is among the few that have documented higher frequency of secondary ameloblastic carcinoma in the scientific literature.

## Introduction

Ameloblastoma is a slowly-growing, locally-invasive epithelial odontogenic tumour and has been regarded as the most common odontogenic tumour in many studies [1-3]. However, other studies have documented it as the second most prevalent odontogenic tumor after the odontomas [4, 5]. It has a reported prevalence of about 1-3% of all jaw tumors and cysts, with varied geographical prevalence ranging from 11-24% of all odontogenic tumors in North America to an estimated global incidence of 0.5 cases per million person-years [6, 7]. Ameloblastoma constituted between 66%-99% of odontogenic tumors in sub-Saharan Africa, with the mandible being more commonly affected (in about 80% of cases) [6, 8]. Ameloblastoma rarely exhibit malignant transformation, as evidenced by the low frequency of occurrence of Ameloblastic carcinoma (AC). More than 100 cases of AC have been reported in the scientific literature, many of which are case reports; hence, there is currently a paucity of information on AC [9-11]. Controversy and lack of consensus still trails AC with regards to its nomenclature and prognosis, due to the relative rarity of this lesion; however, updated classification of Head and Neck tumors by World Health Organization in 2005 seem to have resolved this issue [12]. In this classification, AC was included as one of the odontogenic malignancies. Epithelial malignant odontogenic tumors in this category were: metastasizing (malignant) ameloblastoma (MA), ameloblastic carcinoma (AC), primary intraosseous squamous cell carcinoma (PIOSCC), clear cell odontogenic carcinoma (CCOC) and ghost cell odontogenic carcinoma (GCOC).

Earlier on (in 1982), Elzay *et al.* proposed a classification in which carcinomas occurring within the jaw bones (excluding cases of invasion from the oral mucosa, distant metastasis, and salivary gland) were classified based on the histological evidence of origin thereby describing AC as tumors having the characteristics of ameloblastomas and squamous cell carcinomas [13]. Malignant (metastasizing) ameloblastoma and ameloblastic carcinoma are two distinct malignant variants of ameloblastoma. While AC arises either de novo, from ex-ameloblastoma or from odontogenic cyst [14]; malignant ameloblastoma is entirely an ameloblastoma with probable capacity to metastasis [10, 15]. AC as an odontogenic malignancy, combines the histological features of ameloblastoma with cytological atypia, even in the absence of metastases [13]. It can be further subdivided into the primary and secondary subtypes [8]. The primary subtype, otherwise known as *de novo*, presents malignant neoplasm *ab initio*; whereas, the secondary subtype demonstrates a malignant transformation observed in a previously existing ameloblastoma, independent of the presence or absence of metastasis [8, 16]. There is further division of the secondary type of AC into two subtypes namely the intraosseous type arising within a preexisting benign intraosseous ameloblastoma, and the peripheral type, arising within a benign peripheral ameloblastoma [12]. Metastasizing (malignant) ameloblastoma, is known as a tumour that retains its benign histological features regardless of the site of metastasis [9].

Ameloblastic carcinoma (AC) is very rare, nevertheless it is the most common malignancy of odontogenic tumours (OTs) and presents a higher incidence compared to that of malignant ameloblastoma by a ratio of 2:1 [7, 17]. The clinical features and biologic behavior of AC are seemingly identical to ameloblastoma in terms of associated bony swelling, tooth mobility and multilocular radiographic presentation, nevertheless they can be distinguishable on the basis of thorough clinical examination and associated features such as attendant pain, ulceration, rapid rate of growth and cortical bone perforation with or without soft tissue invasion, which are often suggestive of malignancy [9, 17]. Meticulous microscopic evidence of cytological atypia with presence of mitosis confirms the diagnosis of AC, despite the observation of classical histopathological features of benign ameloblastoma. AC is characterized by diverse histopathological features which presents a diagnostic dilemma, hence proper evaluation of the patients and extensive analysis of the surgical sections of specimen is advocated. The relative frequency of AC is less than 1% of all malignant neoplasms in the Head and neck region (H&N) [18]; and despite its low occurrence, its diagnostic dilemma necessitates highlighting of its essential clinical behavior and microscopic presentation. This is very important in oral pathology practices in sub-saharan regions of Africa, where human and financial resources are limited. Furthermore, advanced studies in immunohistochemistry and genetics can also facilitate precise diagnosis of AC [10]. Currently, there is dearth of report on AC from Africa, and majority of available studies in the scientific literature are cases reports from Asia and America [9-11, 19]. Hence, the present study aims to highlight the clinical features and histopathologic behavior of AC in a tertiary health centre in Nigeria.

## Methods

This study reviewed the clinicopathological data on AC in relation to other odontogenic tumors (OTs) over a 10-year period (2007-2016), from the surgical pathology archive of the Oral Pathology Unit, of the Department of Oral Maxillofacial Surgery and Oral Pathology, Obafemi Awolowo University Teaching Hospital Complex (OAUTHC), Ile-Ife, Nigeria.

**Clinicopathological details:** OTs that was designated as AC in accordance to WHO (2005) classification with adequate information was selected for the study. Information regarding the bio data of the patients (age, gender and site, duration of the lesion, signs and symptoms (specifically swelling, pain, ulceration and cortical bone perforation), radiographic features, type of AC (primary or secondary) and recurrence were obtained from the hospital record and summarized in Table 1.

**Table 1:** Clinic pathological review of 13 cases of meroblastic carcinoma from OAUTHC, Ile-Ife between 2007 and 2016

Case No.	Gender/Age	Type	Location	Signs/ Symptoms	Duration (Years)	X-rays	Margin
1	M/44	S	Maxilla (P-S)	Ulcer + Swelling	11	Lytic	ID
2	M/54	S	Mandible (P-R)	Pain + Ulcer + Swelling	6	Multilocular	ID
3	M/34	S	Mandible (BP)	Pain + Swelling	3	Multilocular	WD
4	F/5	PT	Maxilla (A-P)	Pain + Swelling	4mo	Unilocular	ID
5	M/49	S	Mandible (A-P-R)	Pain + Ulcer + Swelling	4	Lytic	WD
6	M/47	S	Mandible (P-R)	Swelling	15	Multilocular	ID
7	F/60	PT	Maxilla (A-P-S)	Pain + Ulcer + Swelling	2	Multilocular	WD
8	M/32	S	Mandible (BP)	Swelling	8	Multilocular	ID
9	M/32	PT	Mandible (A)	Pain + Swelling	7mo	Multilocular	WD
10	M/46	-	Mandible (A-P-R)	Pain + Swelling	5	Multilocular	ID
11	F/24	S	Mandible (BP)	Pain + Ulcer +S welling	4	Lytic	ID
12	F/16	S	Mandible (BP-R)	Swelling	3	Multilocular	ID
13	F/36	S	Mandible (P-R)	Pain + Ulcer + Swelling	16	Multilocular	ID

**S**, secondary type; **PT**, primary type; **P**, posterior (distal to canine); **R**, involving the ramus; **P-S** ,posterior involving maxillary sinus; **A**, anterior ; **BP**, bilateral posterior; **ID**, Ill-defined; **WD**, Well-defined; **mo**, Months

**Histopathological reports:** Histopathology reports were obtained from biopsy record books of the department. Haematoxylin and Eosin (H & E) stained slides of diagnosed ACs were retrieved for re-evaluation and re-confirmation. Cases that presenting doubtful microscopic observations were revisited by obtaining newly prepared H & E slides from formalin-fixed paraffin-embedded (FFPE) blocks and reevaluated for confirmation of diagnosis wherever necessary. Histopathology features obtained are summarized in Table 2. The histopathology parameters were graded by dividing each of the two slides into four quadrants using a slide pen, this is done for each lesion at the centre of the mounted specimen (at X10 Magnification). A score of 25 was assigned per quadrant based on the parameters (hence, making up 100% for each slide). This score was recorded for individual lesions as follows; occasional finding + (25%); frequently seen ++ (25%-50%); dominant +++ (≥ 50%).

**Table 2:** Histopathological features of 13 patients with ameloblastic carcinoma

Features*	1	2	3	4	5	6	7	8	9	10	11	12	13
Plexiform	+	-	-	+	++	+	-	++	+	+	+	-	-
Follicular	+++	+++	+++	++	+	+++	++	+	++	++	+++	++	+++
Reverse Polarization	+++	++	+	++	+	+	++	++	+	++	++	+	++
Peripheral Palisading	+++	++	++	+	++	++	+++	+++	+	++	+++	++	++
Stellate Reticulum	+	+	++	++	-	+	+	++	-	+	+	-	+
Clear Cells	++	++	++	+++	-	-	++	-	++	+	+	+	++
Ghost Cells	-	-	-	-	-	-	-	-	-	-	-	-	-
Necrosis	+	-	++	+++	+++	++	++	-	-	++	-	++	++
Mitosis	++	+++	+	++	++	++	+	-	++	++	+	++	++
Keratin	-	+	+	+	+	+	+	+	-	+	-	++	+
Basilar Hyperplasia	+++	++	++	+	+	++	+++	++	+	++	+++	++	++
Crowding of cells	+	+++	++	++	++	+	+	++	++	+	+++	+++	+
Invasion (Vascular/Neural)	-	-	+	+	-	++	-	-	+	-	-	-	+

* The presence of each feature is indicated as follows (the corresponding percentage of the tumor applied to all categories except “mitoses”): occasional (25%); frequent (25%-50%);Dominant (50%). Number of mitoses per high-power field: 1-2; 3-4; 5-6

**Quality assurance:** Two independent certified pathologists carried out all slide examination and the average score was taken as the final score for each lesion as summarized above (Table 2).

**Statistical analysis:** Data was analyzed using Stata (MP 13.1 for MacOS StataCorp, Lakeway Dr, College Station, Texas, USA).

## Results

A total of 790 cases of Oral and jaw lesions were seen within the 10-year study period. Of these, 157 (19.9%) cases of OTs were found out of which 102 (64.9%) cases were Ameloblastomas. ACs constituted 13 (1.64%) cases of the total biopsy and 8.3% of OTs. Table 1 summarizes the demography of ACs in this study. Three cases were primary subtypes of AC, constituting 23.08%, while 9 (69.23%) cases where of the secondary subtype. It was observed that the secondary subtypes had a higher frequency of occurrence. One case could not be sufficiently accounted for; hence, it was not categorized either as primary or secondary AC subtype.

**Figure 1:**
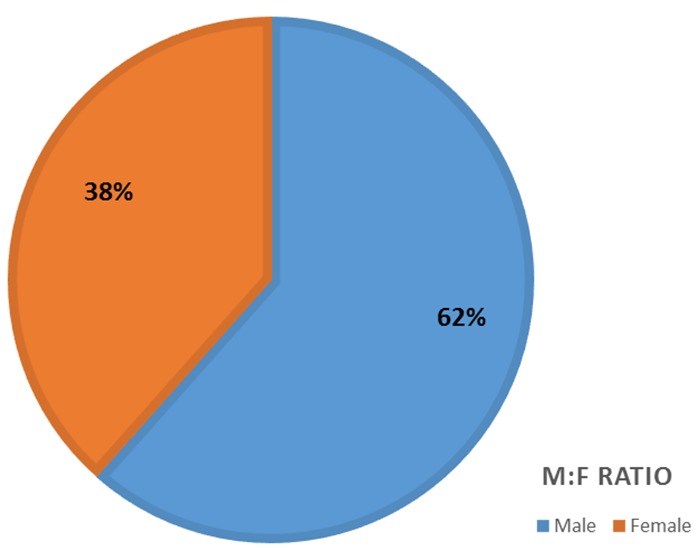
Clinicopathological detail of 13 ameloblastic carcinoma cases. There was a male predominance among all ameloblastic carcinoma cases

**Patient demographics:** The mean age of the 13 cases analyzed in our study was 36.8 years (± 15.5), with the age range between 5 and 60 years, and a peak age incidence in the 7^th^ decade of life. Most of the cases (61.5%) were observed within the 4^th^ and 5^th^ decades of life. Mean age duration for males was 42.25 years (± 8.46) and 28.2 years (± 21.07) for females. Analysis showed male preponderance of 8 (61.54%) with a male: female ratio of 1.6:1 (Figure 1). Mean age for cases in the mandible was 37 years, which is approximately equal to that of the maxilla (36.3years), hence showing no significant difference.

**Duration:** The overall mean duration prior to presentation was about 6years (5.99 yrs), ranging from 4 months to 16 years. The secondary type showed a higher mean duration value of 7.7 yrs.

**Figure 2:**
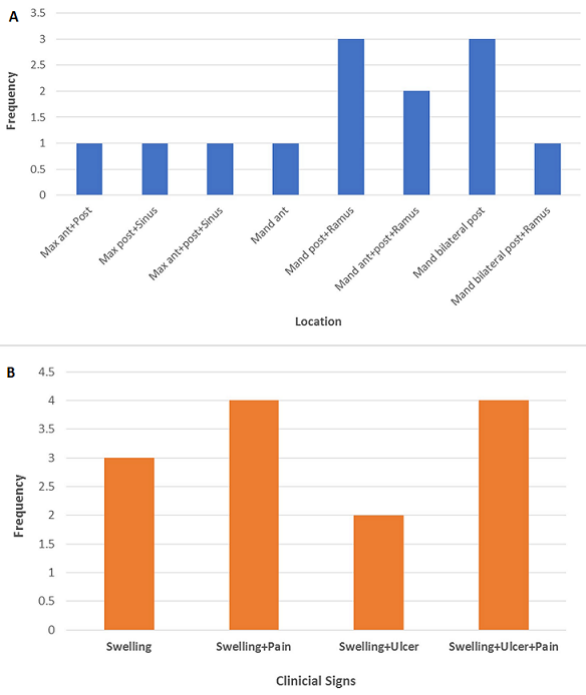
A) the mandible was more affected than the maxilla and posterior region was common for both jaws; B) Also, swelling was a common feature of all cases with additional features such as pain and ulceration

**Location of lesions:** Mandible was the most frequent site of affectation, accounting for 10 (76.9%) cases. We observed a mandible: maxilla ratio of 3.3:1. Furthermore, majority of the cases (69.2%) were seen in the posterior regions of either jaw (Figure 2 A).

**Figure 3:**
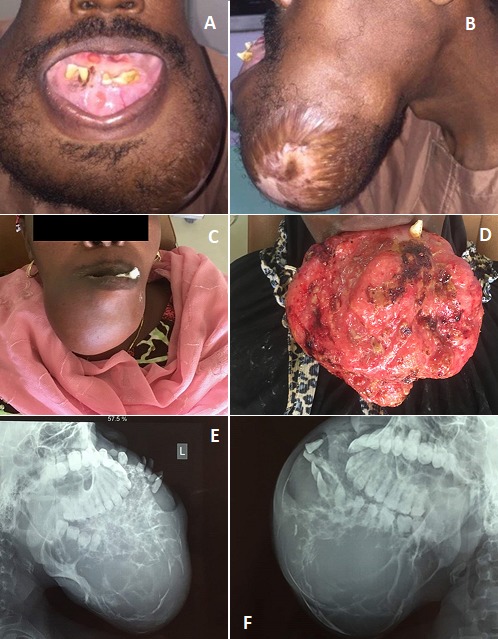
Clinical and radiological features of ameloblastic carcinoma. Ameloblastic carcinoma may presents as an aggressive expansile swelling of the jaws (A-C), and can present an exophytic extraoral mass (D). Radiographically, it can present as a jaw mass will an ill-defined border (E, F)

**Clinical features:** Swelling was consistently seen in all cases (100%), and pain with/without ulceration was present in (61.54%) (Figure 2 B and Figure 3, A-D). Bone perforation was seen in 8 (61.54%) cases and two recurrent cases were seen after the initial surgical treatment.

**Radiographic findings:** Plain radiographs generally showed mixed radiolucency/radioopacity, with more of multilocular appearance in some areas (Figure 3 D, F). Well-defined margin was observed in 4 (30.77%) cases, which was lower than cases with ill-defined borders 9 (69.23%).

**Figure 4:**
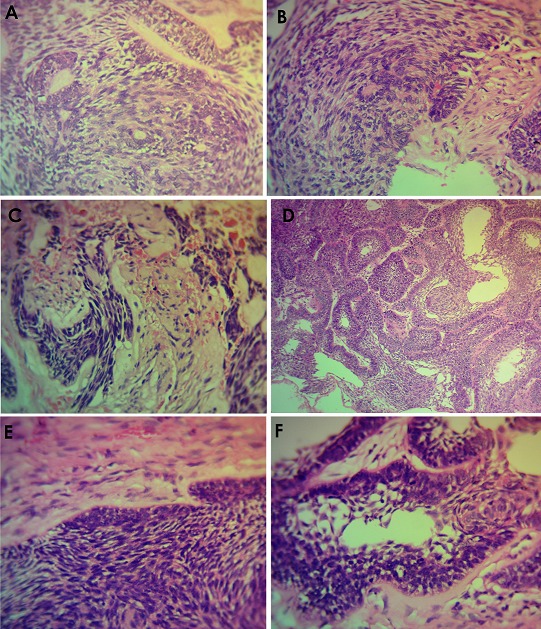
Overview of histopathological finding in Ameloblastic carcinoma. Photomicrographs shows presence of cellular atypia with nuclear hyperchromatism, and cytoplasmic clearing (A). There were areas showing discohesion of the malignant cells, with invasion into the underlying deeper tissue (B). Perivascular and intravascular invasion by the discohesive Ameloblastic carcinomatous cells was observed (C). Also seen were islands of neoplastic malignant transformation of the peripheral basal cells with loss of polarity and presence of mitotic figures, as well as areas of the tumour islands undergoing necrosis (D). There was spindling of the cells with presence of cellular atypia involving the basal cells (E). Also seen were disruption of the basal cells with hyperchromatic nuclei, pleomorphic and clear cells; with presence of squamous cell carcinomatous transformation in the central area (F)

**Figure 5:**
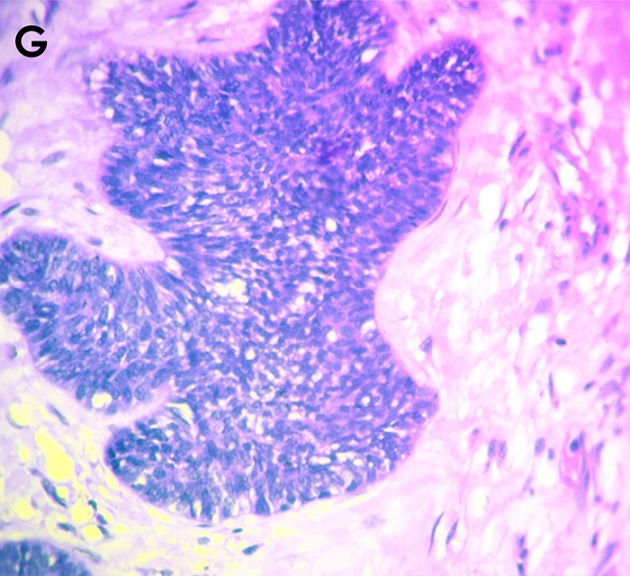
Anaplastic transformation with mitotic cells and hyperchromatic nuclei were also observed

**Histology:** Histologic features mimicking conventional ameloblastoma predominantly follicular growth pattern was seen in about 76.9%, as against 25.6% of plexiform pattern. Presence of peripheral palisading of the basal cell was dominant with 71.8% and reverse polarity of the nuclei were seen in 56.4% of all the cases. There was minimal to low quantity of loose stellate reticulum-like cells (33.3%). Basilar hyperplasia and tumour crowding centrally constituted 66.7% and 61.5%, respectively. Mitotic figures were seen in appreciable areas, constituting 56.4%, while necrosis and clear cells were found in 41.7% and 46.2%, respectively. Keratin pearl formation was seen in 28.2%, while ghost cell was absent in all of our cases. Perivascular invasion was only present in 15.4% of cases. Cytological atypia including pleomorphism, abnormal chromaticity and reverse nuclear/cytoplasmic ratio were frequently observed. Overview of histological finding is provided in Figure 4 and Figure 5.

## Discussion

The term ameloblastic carcinoma (AC) was introduced by Shafer *et al.*, (1974) to describe a tumor arising from malignant transformation of epithelial cells of an ameloblastoma [20]. Despite the rarity of AC compared to ameloblastoma, AC is the most common malignant OT. The pathophysiological mechanism of its evolution is still poorly understood, and this has resulted in a lot of controversy about its biology [11, 19]. Sequential carcinogenesis in ameloblastoma has been suggested to involve long term accumulation of multiple genetic defects. This could also be due to a spontaneous mechanism following repeated therapies which may eventually lead to aggressive clinical manifestation [9, 21]. Primary types of AC have been widely reported to be more common than the secondary type [8, 9], however finding from this study contradicts this observation. We found the secondary type being more frequent with a remarkable ratio of 3:1. Furthermore, the overall mean duration at the time of presentation was 5.9 years for primary AC, as compared with the secondary type which was 7.7 years. The range of occurrence of secondary AC in our study was 4 months to 16 years. This alludes to the fact that most of our cases arose from carcinomatous dedifferentiation of a pre-existing intraosseous ameloblastoma (as evident from the initial documented histologic diagnosis of ameloblastoma). To the best of our knowledge, this is one of the few studies of AC from the sub-Saharan African region where secondary AC is more frequent than the primary type. A possible factor attributable to this is patients' attitude. Most patients tend to delay in seeking orthodox medical treatment (as indicated in the mean year of duration) and there are limited resources for timely surgical intervention. Zarbo *et al.* (2003), submitted that malignant transformation may take up to 10yrs to occur [22], and this is consistent with what we found in our study. Repeated surgical trauma with induction of chronic inflammation was also noted in this study as some of our patients were exposed to multiple biopsies prior to surgery. The high occurrence of AC relative to OTs observed in this study is in contrast with the report from Ladeinde *et al.* (2005) and Jing *et al.* (2007) both presenting an incidence of 2.2% and 1.6% from 391 and 1642 OTs, respectively [7, 17]. There is no evidence yet to ascertain this disparity, however it should be noted that this study retrospectively reviewed cases seen within a decade, in comparison to the previous studies where overall cases of OTs were reviewed [7, 17].

Demographically, AC presents diverse clinical features most especially in terms of age, gender and site [11, 23]. Findings from the literature showed that it occurs in an average age range of 30-50 years [6], which concurs with our finding. Our study recorded a mean age of 36.8 years (± 15.5) with a range of 5-60 year which was comparable to that observed in other studies [23-25]. It is however lower than mean ages recorded in some studies [9, 11, 26, 27], thus confirming the reported variation in the mean age of the patients diagnosed with AC. Dhir *et al.* (2003), in their review of 18 cases of ACs observed a higher mean age for maxillary lesions, as compared to the mean ages reported for mandibular lesions [28]. Their findings suggest that maxillary lesions probably occurs in slightly older patients as compared to mandibular ones. Similar reports were documented by Yoon *et al.* (2009), where a mean age of 56.7 years for maxillary and 49.1 years for mandibular lesions was documented [11]. Although these results were at variance with our findings where maxillary and mandibular cases showed almost equal mean ages (36.3 yrs and 37 years, respectively); this might not be unconnected to the low number of maxillary cases (n=3) in our study. Hence, a larger study could probably substantiate this observation. The gender predilection in our study is predominantly male, although some studies have reported slightly varied findings of which no apparent gender predilection was observed [6, 24]. The general consensus from most studies is higher frequency of occurrence in males [9, 11, 26]. Findings from our study of male to female ratio of 1.6:1 concur with general observation from previous studies. This higher male predilection of AC follows the distribution pattern of its benign counterpart, ameloblastoma [6]. Although mandible is the preferred site for AC as seen in our study, however, a systematic review has shown equal predilection for both jaws [9]. In addition, 69.2% of our cases involved the posterior region of either jaw which is in accordance with other studies [9, 11]. AC presents more aggressive biologic behavior and the most commonly observed characteristics are swelling often with pain, rapid growth and cortical perforation [11, 27]. Trismus, gingival bleeding, paresthesia, epistaxis and dysphonia have also been mentioned [24].

All our cases presented with obvious clinical swelling often associated with pain however, it is pertinent to note that painless swelling can be encountered sometimes as observed in about 38.5% of our cases, and this can masked the suspicion of a malignant lesion. Many studies in the scientific literature are in agreement with our finding of some AC presenting a painless swelling [11, 15, 24, 29]. Other features include rapid growth, perforation of the cortex with ulceration of the soft tissue as observed in 61.5% of cases in this study. Multilocular and sometimes unilocular radiolucency has been described as a common radiographic presentation of AC which is consistent with that of ameloblastoma. However, the margin of AC is often ill-defined with occasional presence of focal radio-opacity [15]. Likewise, this is the most frequent observation in this study, as 69.2% presented with decorticated border. This is also consistent with other reports [24, 30, 31], but contradicts the findings by Yoon *et al.* [11], where 80% presented well defined borders. This therefore portend that AC may demonstrate divergent radiological findings. Advanced imaging technique like CT scan and MRI could provide more precise outcomes; albeit, not many centers in sub-Saharan Africa have access to these instrumentations, routinely. Due to the rarity of AC, precise histopathological diagnostic features have been elusive [20, 32]. However, researchers have highlighted significant microscopic features that could aid in the diagnosis of AC. Akrish *et al*, in their 20 year review of 37 cases of AC, concluded that histologic evidence of ameloblastoma-like lesion with some areas demonstrating cytological atypia (including cellular and nuclei pleomorphism, hyperchromatism, enlarged nucleoli, abnormal and increased mitosis, and mitotic figures) should alert the pathologist to the possible diagnosis of AC [19, 24, 27]. Furthermore, other histological parameters including central cell spindling, minimal presence or lack of stellate reticulum-like area, squamous metaplasia with keratinization, reversal of nuclei polarity, crowding/hyperplasia of the basal cells, necrosis, neural and/or vascular invasion and appreciable presence of clear cells have also been mentioned as essential clues to the diagnosis of AC [11, 12, 24].

As seen in our study, the tumour islands exhibited both plexiform and follicular growth pattern which conforms with observations from previous studies [19, 29, 32, 33]; however, the follicular type was very dominant in our cases which agrees with other studies [19, 29, 33]. Basal cells palisading and reverse polarity of the nuclei were present in all 13 cases. Also present in our AC cases were, nuclear hyperchromatism, spindle-like cells proliferation, crowding of the cells towards the central region, and multilayered basal cell. Furthermore, increased presence of abnormal mitotic cells, cellular and nuclear pleomorphism and hyperchromatism were seen in almost all the AC cases. These findings are consistent with reports in literature [9, 30, 34]. Kamath *et al.* [35], categorized AC into four types based on the predominant histologic cell *viz*; ameloblastic cell type, granular cell type, clear cell type and spindle cell type. All except granular cell type were present in our study. Tumor necrosis was found in 48.7% of our cases as seen in the study by Casaroto *et al.* [29], where they found a total of 25% tumor necrosis and 6.3% ghost cells in 32 cases. Hence, tumor necrosis and presence of ghost cells may be important diagnostic indicators for AC. Although neurovascular invasion were scarce in our study, it does not exclude the diagnosis of AC [24, 27, 35]. The use of immunohistochemistry and genetics has also been known to improve the diagnosis of AC [36, 37]. Yoon *et al.* 2011 [37] compared the expression patterns of immunohistochemical markers in 10 cases of ameloblastoma and 7 cases of ameloblastic carcinoma, with observation that AC showed a statistically significant higher value in the expression of CK18, parenchymal MMP-2, stromal MMP-9 and Ki-67, in comparison to Ameloblastoma.

In another study, Abdelhamed *et al.* [38] investigated the expression of midkine and Ki67 immunomarkers in ameloblastoma and AC. The study established that midKine and Ki-67 expression were significantly higher in AC than in ameloblastoma. Furthermore, use of Ki67 and AgNOR to differentiate AC from ameloblastoma showed that these markers were highly expressed in AC [29]. In addition, Lei *et al.* [39] also attempted to differentiate AC from ameloblastoma and atypical ameloblastoma with the use of SOX2 marker (sex determining region-Y-related high mobility group box 2), which is a protein that is involved in ameloblastic epithelial lineage. The study revealed that SOX2 showed a diffuse and strong nuclear staining pattern with 86.4% specificity and 76.9% sensitivity in AC as compared to the benign counterpart. It can therefore be suggested from the immunohistochemistry studies that differential expression of these markers could be used as an ancillary diagnostic tool for AC. Not least, molecular analysis as used in a study has also highlighted a statistically significant presence of p16 mutation (cyclin dependent kinase inhibitor 2A) in all cases of AC samples as compared to observation in only one sample of ameloblastoma [40]. Therefore, it is presumed that p16 alteration may play a role in the malignant progression of AC. Muller *et al.* investigated nuclear chromosomal ploidy to differentiate between ameloblastomas and AC with reported observation of aneuploid chromosomes in ACs and diploid chromosomes in ameloblastomas [41]. Furthermore, advanced imaging technique using positron emission tomography (PET) and magnetic resonance imaging (MRI) have been used to distinguishing AC from benign Ameloblastoma [42]. In this study, preoperative 18F-α-methyl tyrosine positron emission tomography and magnetic resonance imaging was utilized in the identification of malignant region in AC, although the result was inconclusive.

Because of the rarity of large clinical series and long-term follow-up, treatment protocol for AC is debatable. Some clinicians prefer radiotherapy [28], while others advocate for surgical treatment [13, 24]. Ramadas *et al.*, [43] also considered chemotherapy such as use of cisplatin, adriamycin, and cyclophosphamide to be beneficial in cases of unresectable lesion and as post-surgical adjuvant treatment. AC is considered to be radioresistant by some authors and its use is therefore restricted to pre/post-surgical management, cases not suitable for surgical management, as well as in those that exhibit advanced local or metastatic disease [31]. Wide local excision with about 2cm safety margin (with or without adjuvant radiotherapy and or chemotherapy) has been considered as the mainstay of treatment of AC. Although variable successes have been recorded with radiotherapy or surgery either in combination or alone. Role of chemotherapy in the management of AC is still debatable hence it has not been indicated as a primary treatment mode [9]. The benefit of neck dissection is controversial in the absence of evidence of metastasis [30]. Ion beam therapy has also been employed in the management of AC with proven effectiveness and success, most especially in a recurrent lesion or complex surgical case with proximity to vital structures [44]. Although many targeted precision therapies for odontogenic malignancies are still in clinical trials, BRAF V600E inhibitors (e.g. Vemurafenib) and combination checkpoint inhibitors [45, 46], have also been experimentally used for targeted treatment of ameloblastoma and AC.

## Conclusion

AC is a rare malignant lesion that demonstrates a spectrum of demography, clinicopathologic features and biologic behavior that ranges from indolent/benign to aggressive/malignant, as observed in this study. Due to the rare nature of AC, it often presents diagnostic dilemma to many oral pathologists in resource limited settings. Due to lack of consensus on parameters for the diagnosis of AC, cases should thus be carefully analyzed and evaluated both clinically and histopathologically to detect subtle changes that may predict its aggressive behavior and diagnosis. This study is among the few in the scientific literature that documented higher frequency of secondary AC. In addition, little is known till date of its pathogenesis, although delay in seeking orthodox treatment by patients in low and middle income world setting may be an important contributory factor. Although scientific literatures has overwhelmingly favored the surgical management as the treatment of choice, novel emerging molecular therapies may soon overtake conventional therapies in an era of precision medicine.

### What is known about this topic

- Ameloblastic carcinoma is a rare malignant odontogenic neoplasm;
- Ameloblastoma rarely exhibit malignant transformation to ameloblastic carcinoma;
- Due to its rarity, ameloblastic carcinomas is often misdiagnosed as ameloblastoma.

### What this study adds

- This study reviews a large cohort of odontogenic tumors in sub-saharan Africa;
- We have examine variations in presentation of ameloblastic carcinoma and provided tips on how to delineate it from other odontogenic tumors;
- This study documents higher frequency of secondary ameloblastic carcinoma in our study population.

## Competing interests

The authors declare no competing interests.
